# Neonatal Immunization: Rationale, Current State, and Future Prospects

**DOI:** 10.3389/fimmu.2018.00532

**Published:** 2018-04-04

**Authors:** Elizabeth Whittaker, David Goldblatt, Peter McIntyre, Ofer Levy

**Affiliations:** ^1^Centre for International Child Health, Department of Paediatrics, Imperial College London, London, United Kingdom; ^2^Immunobiology Section, UCL Great Ormond Street Institute of Child Health (ICH), London, United Kingdom; ^3^National Centre for Immunisation Research and Surveillance, Kids Research, Sydney Children’s Hospital Network and University of Sydney, Sydney, NSW, Australia; ^4^Precision Vaccines Program, Division of Infectious Diseases, Boston Children’s Hospital, Boston, MA, United States; ^5^Harvard Medical School, Boston, MA, United States

**Keywords:** neonatal, vaccine, protection, trained immunity, novel adjuvants

## Abstract

Infections take their greatest toll in early life necessitating robust approaches to protect the very young. Here, we review the rationale, current state, and future research directions for one such approach: neonatal immunization. Challenges to neonatal immunization include natural concern about safety as well as a distinct neonatal immune system that is generally polarized against Th1 responses to many stimuli such that some vaccines that are effective in adults are not in newborns. Nevertheless, neonatal immunization could result in high-population penetration as birth is a reliable point of healthcare contact, and offers an opportunity for early protection of the young, including preterm newborns who are deficient in maternal antibodies. Despite distinct immunity and reduced responses to some vaccines, several vaccines have proven safe and effective at birth. While some vaccines such as polysaccharide vaccines have little effectiveness at birth, hepatitis B vaccine can prime at birth and requires multiple doses to achieve protection, whereas the live-attenuated Bacille Calmette–Guérin (BCG), may offer single shot protection, potentially in part *via* heterologous (“non-specific”) beneficial effects. Additional vaccines have been studied at birth including those directed against pertussis, pneumococcus, *Haemophilus influenza* type B and rotavirus providing important lessons. Current areas of research in neonatal vaccinology include characterization of early life immune ontogeny, heterogeneity in and heterologous effects of BCG vaccine formulations, applying systems biology and systems serology, *in vitro* platforms that model age-specific human immunity and discovery and development of novel age-specific adjuvantation systems. These approaches may inform, de-risk, and accelerate development of novel vaccines for use in early life. Key stakeholders, including the general public, should be engaged in assessing the opportunities and challenges inherent to neonatal immunization.

## Introduction

Despite the success of the Millenium Development Goal era from 2000 to 2015, during which the under five mortality rate was reduced by 53%, ~ 2 million infants under 6 months die annually due to infections (1). Of the 5.9 million children under 5 years of age who died in 2015, 45% were in the first month of life (2). Many of these deaths are attributed to vaccine preventable illnesses, occurring before protection is afforded by routine immunization given as part of the expanded program of immunization (EPI). Although this commences at 6–8 weeks of age, the first dose does not provide immediate protection and multiple doses are required, leading to vulnerability in the first 6 months of life. In an effort to reduce the under 5-year old mortality rate further, to ≤25/1,000 live births by the end of 2030, a number of strategies are being explored and implemented as part of the sustainable development goal-3. These include maternal immunization, which, although it shows great promise for a number of pathogens, including pertussis and influenza, is limited by safety and ethical concerns, and is of limited value for the ~2.6 million infants born preterm, prior to maternal antibody (Ab) transfer (3). The reality, that we rely on immunization occurring early in life, coupled with recent advances in our understanding of neonatal immune responses (4–6), has led to renewed interest in neonatal immunization as a promising and effective strategy, to reduce morbidity and mortality in young infants. Thus, the topic of early life immunity, and in particular neonatal immunization, is one of tremendous public health relevance.

Great strides in vaccine development over the last century have resulted in a number of effective vaccines being given in early life, but only Bacille Calmette–Guérin (BCG), hepatitis B (HBV), and polio vaccine [oral polio vaccine (OPV); or inactivated polio vaccine (IPV)] have been routinely recommended at birth. For some pathogens, including pertussis and tuberculosis (TB), better vaccines are needed, while for others such as human immunodeficiency virus (HIV) and respiratory syncytial virus (RSV), efficacious vaccines have yet to be developed and licensed for any age group. Among the approaches to improving protection against infection in early life, neonatal immunization is ripe for further research and development. Herein, we review the rationale for neonatal immunization and highlight essential research areas, including the study of immune ontogeny and the development of vaccines optimized for early life administration.

### Rationale for Use of Vaccines in the Neonatal Period

The neonatal period is defined as the first 28 days of life. For the purpose of this review, we define neonatal vaccines as those given “at birth” or within the first 28 days of life. Of note, EPI vaccines are licensed to be given within the first few weeks of life, and in reality, the “birth dose” is given across a range of time in the first month of life, a variability that to our knowledge has not been systematically studied with respect to relative vaccine efficacy. In contrast, we define infant vaccines as those given *after* the first 28 days of life. In countries following the EPI schedule, after the neonatal doses of BCG, HBV, and polio vaccines, the next EPI schedule dose is typically given between 6 and 8 weeks of life. As with any vaccine approach, development of neonatal vaccines must take into account potential limitations, including: (a) need to establish safety, (b) lack of effectiveness of some vaccines in early life, (c) challenges of a translational path that typically starts with formulations optimized for adults, rather than generating formulations that are optimal for the young, and (d) potential blunting of neonatal Ab responses after maternal immunization. Nevertheless, the rationale for neonatal immunization is robust and includes: (a) the heavy burden of early life infection; (b) that birth is a practical point of healthcare contact, and pairing immunization with birth may lead to health benefits for both mothers and newborns; (c) immunization at birth may provide earlier protection than existing immunization schedules; (d) the likely benefit of protection to babies born preterm for whom maternal Ab transfer is limited, with an increased risk of serious infections throughout childhood (7); and (e) emerging evidence that the heterologous benefit of the live-attenuated BCG vaccine and other live vaccines may be greatest in early life (8).

## Lessons from Immune Ontogeny

Neonatal immunization occurs in a backdrop of distinct early life immunity. Recent reviews have highlighted that both cellular and soluble aspects of the immune system are distinct at birth (9, 10). Neonatal immunity must not only defend the newborn against a potential onslaught of potential pathogens, but also mediate the acquisition of a colonizing microbiome over the first hours and days of life. In this context, neonatal immune responses are apparently designed to avoid excessive inflammation with a generally reduced production of pro-inflammatory and Th1-polarizing cytokines to microbial components/pattern recognition receptors (PRR) agonists. Age-specific composition of soluble and cellular factors shape neonatal immunity. The distinct composition of human newborn cord blood plasma includes soluble mediators such as maternal Abs, high levels of immunosuppressive adenosine, and low levels of complement, important for triggering adaptive immune responses (11). Accordingly, modeling age-specific immunity *in vitro* should take into account distinct composition of age-specific autologous plasma, rather than, for example, fetal bovine serum (9). Distinct cellular immunity in the newborn includes reduced Th1 but robust anti-inflammatory IL-10 responses of antigen-presenting cells to stimulation by PRR agonists, high frequency of naïve- and regulatory-T cells and CD71+ erythroid precursors that may limit, for example, responses to pertussis immunization (10, 12, 13). Nevertheless, neonatal immunity is capable of mounting antigen-specific effector responses, as demonstrated by BCG-specific IFNγ production following vaccination at birth (14). Overall, detailed study and modeling of age-specific human immunity may help inform development of vaccine formulations, with or without adjuvants as needed, that may trigger a protective immune response in early life.

## Proof of Concept: Routine Neonatal Vaccines

### Bacille Calmette–Guérin

Bacille Calmette–Guérin is a live-attenuated strain of *Mycobacterium bovis*. Given in areas with high-endemic TB to prevent disseminated TB in infancy, BCG is the most commonly given vaccine with ~4 billion doses administered to date. Although it has been administered for nearly 100 years, several key issues regarding BCG have emerged, including: (a) lack of a clear correlate of protection (CoP); (b) marked heterogeneity between licensed BCG formulations (15); and (c) growing evidence that BCG has heterologous (“non-specific”) beneficial effects, particularly when administered in newborns (16). It is hypothesized that these non-specific benefits may protect against unrelated infections, supporting the use in neonates, beyond any protection against TB infection or disease. Furthermore, association studies suggest that early immunization with BCG-containing regimens may protect against leukemia, allergy, and childhood diabetes among others, possibly *via* heterologous trained immunity (17–19).

A CoP is an immune measure that corresponds to vaccine-induced protection from disease (20). Despite substantial efforts to characterize classic adaptive immunity, including multiple studies of polyfunctional CD4 T cells, a clear CoP for BCG has yet to be established (21). Indeed, increasing evidence that BCG induces trained immunity—i.e., enhanced subsequent innate responses to a range of stimuli (8)—raises the possibility that these innate immune enhancing effects may not only underlie heterologous (non-specific) benefits of BCG vaccine, but may also contribute to, or conceivably be, the major factor in the so called “specific” effect of BCG, i.e., protection against early life TB.

A critical issue with respect to BCG immunization is marked variability between vaccine formulations produced in different production facilities, with the result that “BCG” is not a single entity. After its original manufacture in the Pasteur Institute (Paris, France) in 1921, BCG was shipped to 20 different international sites where the vaccine was repeatedly subcultured under different conditions. This has resulted in diverse licensed BCG formulations that are distinct both by content of live mycobacteria as well as genetic composition. These strains have been shown to have differing immune responses and furthermore, the clinical relevance of this has been illustrated in comparative studies, which suggest, for example, that BCG-Denmark and BCG-Japan may have greater benefit in reducing TB disease than BCG-Russia (15, 22).

Much remains to be learned regarding mechanisms underlying BCG-induced protection. As a complex vaccine comprised of live-attenuated mycobacterium, BCG engages the innate immune system *via* PRRs. Analogy to *M. tuberculosis* as well as direct human *in vitro* studies suggests that BCG may activate *via* multiple toll-like receptors (TLRs) including TLRs-2, -4, -7, -8, and -9 as well as C-type lectin receptors and NLRs (23). Studies of BCG-immunized adults demonstrate a re-programing of monocyte precursors such that the higher expression of PRRs and greater reactivity to stimuli such as TLR agonists. This innate immune enhancing effect of BCG is reminiscent of the effect of administration of TLR agonists to neonatal mice that enhanced innate immune responses, including cytokine induction and phagocyte recruitment, and improved bacterial clearance and survival in a model of neonatal sepsis (24). Such innate immune enhancing effects of prior stimulation have been termed “trained immunity” reflecting an adaptive arm of innate immunity that is noted in plants, insects, and mammals (25). That BCG heterologous (non-specific) benefits are greater in early life (26) suggests ontogeny of underlying immune mechanisms, potentially including trained immunity (27).

### Hepatitis B Vaccine

Hepatitis B vaccine is an alum-adjuvanted vaccine containing hepatitis B surface antigen (HBsAg). The alum-adjuvanted HBV is given within the EPI (Table 1) and also in Australia, Europe, and United States, where a birth dose is recommended (28). With respect to innate immune activation, while the Alum adjuvant present in HBV may engage the inflammasome, HBsAg also interacts with CD14 to activate dendritic cells (29). Importantly, there is a measurable CoP for HBV, namely the titer of anti-HBsAg Abs. Although this CoP has been defined, as with many vaccines, it is not fully understood how HBV vaccine induces protective immunity, as a newborn dose is protective, despite only ~30–50% of newborns responding with “protective” titers after a single dose. This observation suggests that additional mechanisms, including cell-mediated immunity (CMI), may contribute to protection (30). Ab and T cell responses to HBV given to infants are distinct from those of adults, in that infants produced markedly higher serum anti-hepatitis B surface (HBs) Ab titers in one study, and low-Ab levels were associated with lower HBs Ag-specific IFNγ responses and a more Th2-polarized memory response to HBsAg at 1 year (30). Genetic factors, low-birth weight and low-Apgar scores were risk factors for poor HBV response in a study of twins in China (31). Haplotype analysis of Gambian infants suggested that *CDC42, IL19*, and *IL1R1* genes associated with peak anti-HBsAg Ab level (32). Much remains to be learned regarding how HBV protects in early life.

**Table 1:** Immunizations given at different ages.

	Vaccines licensed	Vaccines tested	Future vaccine targets
Pregnancy	aPertussis Tetanus Influenza	(RSV) GBS	Group B *Streptococcus* HIV Malaria
Birth	OPV HepB BCG	DTaP Hib PCV Malaria (e.g., RTS,S/ASO1/2) Recombinant BCG vaccines (e.g., VPM1002) HIV (phase I/IIa) Rotavirus	RSV Salmonella ETEC ncHI Malaria
Infant doses Age 2–4 months	DTaP and DTwP IPV Hib HepB PCV MenB MenC Rotavirus	Malaria (e.g., RTS,S/ASO1/2, and Spf66) Recombinant BCG vaccines (e.g., VPM1002) Novel TB candidates (e.g., MVA85A) HIV (phase I/IIa)	RSV Men ACWY Salmonella ETEC ncHI Malaria
Infant doses Age 12–13 months	Hib PCV MMR MenB MenC Varicella	LAIV	

*RSV (respiratory syncytial virus), Hib (haemophilus influenzae B), BCG (Bacille Calmette–Guerin), OPV (oral polio vaccine), IPV (inactivated polio vaccine), HepB (hepatitis B), DTaP (diphtheria, tetanus, acellular pertussis), DTwP (diphtheria, tetanus, whole cell pertussis), PCV (pneumococcal conjugate vaccine), HIV (human immunodeficiency virus), ETEC (enterotoxic *Escherichia coli*), ncHI (non-encapsulated *Haemophilus influenzae*), Men B,C, ACWY (meningococcal B,C, ACWY), MMR (measles, mumps, rubella), TB (tuberculosis), LAIV (live-attenuated influenza virus). ( ) indicates Trial in progress*.

### Polio

A birth dose of OPV has been recommended by the World Health Organization since 1984. It is hypothesized that a birth dose of OPV may induce mucosal protection prior to colonization or infection with enteric organisms which may interfere with the immune response to doses given later in life. Data on seroconversion following this individual dose of trivalent OPV (tOPV) vary greatly, from 10 to 15% in India to 76% in South Africa, however, the positive impact on levels of neutralizing Abs and seroconversion rates on completion of the routine immunization schedule are undisputed (33). A systematic review of 5,257 infants given tOPV at birth, found that the percentage of newborns who seroconverted at 8 weeks ranged between 6 and 42% for poliovirus type 1, 2 and 63% for type 2, and 1 and 35% for type 3 (34). In addition, there were four studies of IPV in newborns with a seroconversion rate of 8–100% for serotype 1, 15–100% for serotype 2, and 15–94% for serotype 3, measured at 4–6 weeks of life. No serious adverse events related to OPV or IPV doses at birth were reported in these studies, including no cases of acute flaccid paralysis. Some groups have advocated a shift to using IPV because (a) tOPV has been associated with rare cases of vaccine-associated paralytic poliomyelitis (~2–4 cases/million), (b) concerns about the use of live vaccines in immunocompromised individuals, including those with HIV infection, and (c) potential risk of strain reversion. Of note, however, some studies have suggested that similarly to BCG vaccine, a birth dose of live OPV may induce heterologous (“non-specific”) beneficial effects (35). Further research is warranted prior to replacing OPV with IPV (36).

## Clinical Studies of Other Vaccines at Birth

### Pertussis

Initial studies of the role of a birth dose of whole cell pertussis vaccine demonstrated low-Ab titers at 4 months, although there were no randomized studies at that time comparing a birth dose with a dose at 6–8 weeks of age (37). Further studies of neonatal whole cell pertussis immunization were deterred by the suggestion in 1965 that immunization with the whole cell pertussis vaccine combined with diphtheria and tetanus toxoids (DTwP) within 24 h of birth may introduce “immune paralysis” (38). Twenty years later, comparison studies of a birth dose of DTP with routine immunization at 2 months demonstrated significantly lower titers to pertussis toxin (PT) at 9 months of age, and an inverse correlation between cord Ab titers and infant responses (39). Safety concerns about the whole cell vaccine led to a switch to acellular pertussis vaccines (aP, or DTaP) in the 1990s. Initial neonatal studies of aP vaccines, both with and without diphtheria and tetanus antigens, were promising (40, 41), but a conflicting later study showed poorer responses (42). Reports of bystander interference resulting in lower *Haemophilus influenza* type B (Hib) vaccine and HBV responses were concerning (41). A pilot study of the GSK monovalent aP vaccine at birth and 4 weeks demonstrated significantly higher IgG Ab against pertussis antigens at 2 months of age, without reducing subsequent pertussis Ab responses. A larger study of doses at birth and 6 weeks, including influence of maternal immunization, on Ab responses up to 5 years of age is ongoing (43, 44). Follow-up of children from the initial pilot study to 4 years of age demonstrated higher cytokine responses to pertussis antigen stimulation in those who received a birth dose compared with controls at 2 years of age (44). These observations were similar to those in a long-term follow-up study of children vaccinated at birth which showed increasing CMI, as measured by lymphoproliferative capacity, compared with controls (45). As the role of maternal immunization with pertussis becomes more established, it is crucial to include the effect of maternal interference in studies, as even pre-pregnancy immunization may influence later-born infant responses (46). Englund et al. demonstrated a lack of maternal Ab interference on infant immunizations given at 2 months—that is, the PT Ab response to DTaP, unlike DTwP, was not affected by pre-existing Ab to PT (47). Whether this observation also holds true for birth doses requires future study. Given current concerns of waning immunity to aP (48), novel pertussis vaccine formulations, potentially including developing pertussis vaccines that are safe and effective in newborns, are needed to induce robust and durable immunity against this pathogen.

### Haemophilus Influenza Type B

The role of a birth dose of Hib vaccine was explored by a number of groups following the initial success of its introduction into the EPI. Three different conjugate vaccines tested {HIB polysaccharide conjugated to tetanus toxoid, Hib polysaccharide conjugated to a genetically modified diphtheria toxin (HbOC), and Hib polysaccharide conjugated to a *Neisseria meningitidis* outer membrane protein [HbOMP; subsequently noted to be a TLR2 agonist (49)]} all resulted in significantly higher PRP Ab levels at 2 or 4 months compared with controls, suggesting neonatal priming was possible (50, 51). However, these higher Ab levels did not persist for HbOC and declined more rapidly than controls for HbOMP, leading to concerns about waning protection. Further studies have not been undertaken, as epidemiological studies have demonstrated a protective effect of herd immunity on early life burden of invasive Hib disease, presumed due to reduction of asymptomatic nasopharyngeal carriage of Hib among vaccines (52, 53). Conjugate vaccines, through a T cell-dependent immune response, result in very high-protective Ab responses in infants of all ages, which results in reduction of carriage. Furthermore, the licensed 10 valent pneumococcal conjugate vaccine (PCV10; PHid-CV), uses a *Haemophilus* outer membrane protein (protein D) as its carrier protein, and immunization with this reduces the incidence of all invasive *Haemophilus* spp. disease, including non-typable or non-encapsulated *Haemophilus influenzae* (ncHI). This may be of particular importance as invasive disease due to ncHI is commonest in the first month of life (54, 55), so the potential role of the PHid-CV pneumococcal vaccine or a specific ncHI vaccine in neonates should be studied.

### Pneumococcal Conjugate Vaccine

To date, only two published trials have assessed effects of a neonatal dose of the seven valent PCV7 (56, 57). In Kenya, 300 neonates were randomized to receive PCV7 at birth, 10 and 14 weeks or at 6, 10, and 14 weeks (EPI schedule). The safety of giving vaccine at birth was an important endpoint in the study and the researchers saw no significant difference in safety events between the two groups. Serotype-specific IgG binding was measured following the completion of the primary infant schedule at 18 weeks of age. The proportion of infants who had an Ab concentration above the accepted protective threshold of >0.35 μg/mL was similar between the two groups. When a higher threshold (>1.0 µg/mL) was used, proportions above 1 for serotypes 4, 18 C, 19 F were lower in the neonatal group. Geometric mean concentrations of IgG for four serotypes (4, 9V, 18 C, 19 F) were lower in the neonatal group compared with the EPI group at 18 weeks of age. In contrast, the mean avidity indices were significantly higher in the neonatal group for three of the four serotypes tested (4, 6B, 19 F) (57). In both groups, maternal IgG measured in cord blood inversely correlated with the GMC at 18 weeks of age with high-serotype specific cord blood levels associated with lower responses to vaccine. At 9 months of age, 5 months following the third PCV dose, there was no difference in the percentage of infants with an Ab concentration above either the 0.35 or 1.0 µg/mL thresholds. The GMC of serotype 4 specific IgG remained lower and serotype 19 F avidity index remained higher in the neonatal group. Responses to a booster dose of PCV given at 9 months of age and measured 2 weeks later were comparable between the two groups suggesting that there is no tolerance induced by the neonatal dose. Carriage was measured at 18 and 36 weeks in this study with no significant differences detected between the groups.

In a trial undertaken in Papua New Guinea (PNG), 318 infants were randomized to receive either PCV7 at birth, 1 and 2 months, PCV7 at 1–3 months, or no PCV7 (56). Local reactogenicity rates were generally low although higher rates were seen in the infant than the neonatal group. There were no differences in the illness episodes or serious adverse events. At 2 months of age, serotype-specific GMCs were significantly higher in the neonatal group than in the infant group for four of the seven serotypes in PCV7 (4, 9V, 18 C, 19 F). At this point, the neonatal group had received two doses of PCV compared with one dose in the infant group. By 4 months of age, following three doses of the vaccine, GMCs were significantly higher for all serotypes in the infant group than in the neonatal group although 2 months had elapsed since the neonatal groups third dose, a gap that was only 1 month for the infant group. Comparable responses were seen following a pneumococcal polysaccharide vaccine administered at 9 months of age and responses in both PCV7 primed groups were significantly greater than responses in those who were not primed. Nasopharyngeal swabs were collected at ages 1–4 weeks and 3, 9, 18 months, and middle ear discharge if present. The prevalence of pneumococcal carriage was 22% at 1 week of age, rising to 80% by age 3 months and remained >70% thereafter (58). There were no significant differences in PCV7 serotype carriage between PCV recipients and controls at any age (22 vs. 31% at 9 months, *p* = 0.2). At age 9 months, the prevalence of non-PCV7 serotype carriage was 17% higher in PCV7 recipients (48%) than in controls (25%, *p* = 0.02). The authors attributed the limited impact of neonatal or accelerated infant PCV7 schedules on vaccine serotype carriage to the early onset of dense carriage of a broad range of pneumococcal serotypes.

A prior report from the same PNG study examined whether a neonatal PCV7 dose might induce immune tolerance (59). In a comprehensive immuno-phenotypic analysis at 9 months of age, no differences in the quantity or quality of vaccine-specific T cell memory responses (including responses to CRM197, tetanus toxoid, and HBsAg) were found between the neonatal and infant vaccination groups. Hospitalization rates in the first month of life did not differ between children vaccinated with PCV at birth or not. Reviewing the data outlined in these two studies demonstrates that neonatal immunization with PCV7 is safe and not associated with immunologic tolerance (56, 58).

## Current Research on Early Life Immunization

### Enhancing Current Vaccines

One approach to developing enhanced neonatal vaccines focuses on improving existing vaccines such as the live “self-adjuvanted” BCG vaccine. For example, the BCG-derivative VPM1002 expresses listeriolysin from *Listeria monocytogenes* designed to enhance MHC-I responses (60). In a phase II open label study comparison with conventional BCG-SSI in South African newborns (*n* = 48), VPM1002 demonstrated safety and immunogenicity with an increased proportion of CD8+ IL-17+ cells at 6 months post-vaccine. The authors speculate that although the significance of such cells is unknown, it is possible that they could contribute to more robust protection against TB and that larger studies are needed to assess this possibility.

### Development of Adjuvants for Early Life Immunization

Another approach to enhancing vaccine responses in infants with “age-appropriate” immunity is the addition of adjuvantation systems to enhance vaccine immunogenicity and efficacy. PRR agonists such as mono-phosphoryl lipid A that activates TLR4, have been employed as vaccine adjuvants but the translational path for this approach must take into account that responses to PRR stimulation vary markedly with the age of a given individual (6). In developing adjuvant systems optimized to early life, there may be lessons to learn from live-attenuated vaccines currently in use. Examples from human *in vitro* studies using the BCG vaccine demonstrate this, including TLR2-mediated activation of neonatal NK cells to produce IFNγ; TLR9-mediated activation of pDCs for IFNγ; TLR2- and IFN-mediated activation of conventional DCs IL-12 p70 production and subsequent CD4+ T-cell Th1 polarization (61). Along these lines, TLR7/8 adjuvant-containing nanoparticles mimic effects of BCG on human neonatal monocyte-derived DCs *in vitro* and induce anti-mycobacterial T cells in humanized TLR8 mice *in vivo* (62). Addition of a TLR7/8 agonist adjuvant to PCV dramatically accelerated and enhanced responses to a birth dose, inducing pneumococcal-specific Ab titers far exceeding the CoP after a single dose (63). As with any vaccine development, safety will be front and center, and in this context, it is worth noting that the most commonly given neonatal vaccine, BCG, activates multiple PRRs including TLRs and is safely given to newborns across the globe. Although this does not necessarily imply that any TLR-stimulating approach would be safe, it does provide an important proof of concept that in certain settings and contexts, PRR activation including TLR-stimulation can be safe and effective approach to vaccine adjuvantation in human newborns.

### Mucosal Vaccine Development

In designing new vaccines to be given in the neonatal period, consideration must be given to ideal characteristics (64). Mucosal vaccines, with their potential for needle-free delivery, are very attractive. A number of the pathogens causing severe disease in early infancy are mucosally transmitted [rotavirus, RSV, polio, non-typable *Salmonella* spp., enterotoxic *Escherichia coli* (ETEC)] and the positive experience with OPV provides proof of concept of mucosal vaccines in this age group. Mucosal vaccines could be administered by a number of routes (oral, nasal, conjunctival, rectal, and vaginal) with nasal and oral being the most practical and studied to date. Adjuvanted mucosally administered vaccines stimulate multiple types of immune responses, including secretory IgA Abs which prevent adhesion and invasion of pathogens, serum IgG neutralizing Abs, and a wide array of cell-mediated T cell responses (65). Of note, determining the correlate of immune protection for each vaccine is a challenge. The focus has historically been on neutralizing IgG antibodies, but increasingly, the role of antigen-specific IgA in serum and stool has been explored (66) with need for further standardization of these assays. A number of factors may influence the efficacy and immunogenicity of oral vaccines, particularly in early life, including the presence of pre-existing Abs, malnutrition, enteropathy, micronutrient deficiencies, and breast feeding, factors being studied in the context of OPV and rotavirus vaccines (67). Breast milk contains an array of protective molecules including specific Abs, oligosaccharides, glycoproteins, and receptor analogs, likely to prevent both pathogenic- and vaccine-strains of micro-organisms binding to the intestinal wall. A study of Rotarix in infants comparing withholding or not withholding breastfeeding around the time of vaccine administration did not demonstrate an increase in anti-rotavirus IgA seroconversion (68). To the extent that these factors may limit responses to mucosal vaccines, neonates, who are unlikely to yet have malnutrition, enteropathy, and co-infections, may be more responsive to oral vaccines.

A number of oral vaccines with varying immunogenic and protective efficacies have been licensed including those directed against typhoid, rotavirus, polio, and cholera. Other targets include non-typable Salmonella, ETEC, Shigella, and adenovirus (64). Few to date have been studied in newborns, although diarrheal illness accounts for many of the deaths in infants <6 months of age, with rotavirus identified as a leading cause of dehydrating gastroenteritis, associated with ~28% of diarrheal deaths (69). Two live-attenuated oral rotavirus vaccines, the pentavalent human-bovine rotavirus vaccine, RotaTeq, given as three oral doses a month apart; and monovalent human rotavirus vaccine Rotarix, given as two doses a month apart; were licensed in 2006. Protection against severe disease in high- and middle income countries is excellent for both (80–95% efficacy). However, unfortunately, these rotavirus vaccines have been less efficacious in low-income regions where the need is far greater such as sub-Saharan Africa (46% efficacy) and in Southern Asia (50%) (70). Although indirect benefits of rotavirus vaccine, such as those realized *via* herd immunity and protection, have been described in high- and middle income countries, this has not been firmly established in low-income countries to date (71). Factors including breastmilk Abs, concurrent infections, or environmental enteropathy may interfere with the efficacy of the oral rotavirus vaccine (67). Administration of a birth dose could potentially ameliorate these concerns, as well as provide protection in the vulnerable gap when the most severe disease occurs, between birth and the protective response induced by a first dose given at 6 weeks of age (72). Furthermore, the risk of intussusception as a complication of rotavirus vaccine appears to follow an age-related pattern, supporting a neonatal schedule over an infant schedule for this vaccine (73). A phase IIa study of a monovalent human rotavirus vaccine RV3-BB, including a neonatal dose, demonstrated a rotavirus IgA response rate of 11% with stool excretion of 13% after one dose (74). Overall immunogenicity following the non-neonatal schedule at 8, 15, and 24 weeks was 50% after two doses and 74% after 3. A nested study within the trial examined the relationship between rotavirus-specific IgA in cord blood, colostrum and breast milk and infant serum IgA response and stool excretion and found no evidence of an association (75). Although these initial immunogenicity results are disappointing, with low uptake following the neonatal dose, neither RotaTeq nor Rotarix have been tested in the neonatal period such that further studies of rotavirus vaccines in the neonatal period are warranted.

A nasally delivered live-attenuated influenza vaccine (LAIV) has been effective in infants in protection from flu, but has not to date been tested in neonates. Infants between 6 and 12 months experienced relatively high rates of hospitalization in an RCT comparing inactivated vaccine and LAIV and consequently, LAIV has only been recommended in infants greater than 12 months of age (76). There is currently no available influenza vaccine for infants younger than 6 months, however, maternal immunization provides passive protection (77) and in a murine model, there was no evidence of maternal interference (78).

As a leading cause of morbidity and mortality in the neonatal and early infancy period, RSV is an ideal target for a mucosal vaccine (79). The RSV fusion (F) surface glycoprotein has been considered as one of the two major protective antigens for eliciting neutralizing Abs; a humanized monoclonal Ab specific to the F protein (Palivizumab), administered monthly to vulnerable infants during the RSV season, is efficacious in preventing severe disease, but not infection (80). Low levels of nasal RSV-specific IgA are a risk factor for RSV disease in adults (81), but as yet, although animal models have demonstrated neutralizing Abs in response to RSV vaccine candidates, specific IgA production has not been shown (82, 83). Animal models of live-attenuated vaccines provide grounds for optimism (84) and phase I studies of virus-vectored vaccines in adults have been promising (85), but virus-vectored RSV vaccines have not yet been trialed in infants. Development of vaccines based on the RSV pre-fusion protein, an antigenic target to which protective neutralizing Abs in human sera are directed (82), offers fresh avenues for RSV vaccine development with much to be learned regarding the potential of this antigen when administered in early life.

### Systems Biology

Systems biology approaches have been applied to adults and older infants but thus far not to newborns (86). This deficiency is to soon change with the award of a NIH Human Immunology Project Consortium grant to employ systems vaccinology to study HBV-induced molecular signatures in relation to CoP—i.e., anti-HBsAg Ab responses. Led by the Expanded Program on Immunization Consortium, an affiliation of Boston Children’s Hospital, Medical Research Council (UK)-Gambia, and the University of British Columbia, focused on application of systems biology to early life vaccinology, this study will leverage transcriptomics and proteomics to provide fresh insights into HBV-induced protection in newborns. Among the novel systems biology approaches is “systems serology” that offers potential for much more precise understanding on the impact of vaccines at different ages measuring all aspects of the response, Ab avidity, titer, specific, and non-specific responses (87). This approach promises to provide deeper insight into the vaccine-induced humoral immune responses of distinct populations such as human newborns.

### *In Vitro* Modeling to Accelerate and De-Risk Early Life Vaccine Development

Vaccine development is inherently costly, slow, and unfortunately beset by multiple failures. Current paradigms of vaccine development tend to presume that all populations will respond similarly to a given formulation and do not take species-specificity, genetic background, and age-specificity into account. Accordingly, many vaccine formulations fail or are less effective in vulnerable sub-populations such as the very young or elderly. In this context, human *in vitro* platforms that model age-specific vaccine-induced innate and adaptive immune responses as benchmarked to licensed vaccines offer the possibility of accelerating and de-risking vaccine development (9, 88). Indeed age-specific human *in vitro* platforms such as newborn whole blood assays, dendritic cell arrays, and microphysiologic tissue constructs (89) have been successfully used to define novel biomarkers of vaccine adjuvanticity (90) and identify TLR7/8 agonists as adjuvants active in early life (63).

Overall these technical advances offer powerful new opportunities to inform, de-risk, and accelerate novel vaccine development for use in early life.

## Integration into Current Program: Challenges and Strategies

Neonatal immunization carries tremendous potential but further expansion or enhancement of this approach will require both deeper mechanistic insight into how vaccines protect in early life as well as integration into the existing framework of public health, including maternal immunization programs. In response to the concerns of multiple professionals in the field of maternal immunization, the Brighton Collaboration was formed in 2000, followed by the formation of the Global Alignment of Immunization safety Assessment in pregnancy (GAIA) to establish safety and efficacy standards in this area (91). A collection of case definitions and guidelines for data collection, analysis, and presentation of safety data in vaccine trials, relevant for neonates and infants, were published by global experts, largely in the context of maternal immunization, but these are also relevant and of value for monitoring safety of neonatal immunization studies (92). Whilst these definitions and guidelines can be amended for the use in neonatal immunization studies, the formation of an independent group is warranted to establish the framework for safe and efficacious neonatal immunization studies.

In 2010, to identify key topics and research gaps in the field and foster collaboration among investigators focusing on vaccinology and immune ontogeny, a workshop was organized by NIH (NIAID; Division of Allergy, Immunology, and Transplantation) and cosponsored by the Bill and Melinda Gates Foundation (93). Given recent technical and conceptual advances, and their potential to vastly transform the area of early life immunization, further workshops on optimizing early life immunization, including those individuals involved in regulation and safety assessments are warranted. The study populations for any ongoing vaccine trials should include not just healthy term infants, but a number of distinct populations, including preterm infants and those with immunodeficiencies, in particular HIV-exposed infected and uninfected [but not unaffected (94)], to ensure protection of all infants, especially the most vulnerable. Thoughtful design of studies, both of maternal and neonatal vaccines, will be essential to understand mechanisms underlying vaccine–vaccine interactions, including interference. It is essential that whilst adopting the most advanced systems-based approaches, the data are standardized to allow comparison of sample sets from the same or different sites. Ongoing reassessment of infant immunization schedules will allow the development of more effective neonatal vaccine schedules.

## Conclusion

Overall, neonatal immunization is a common practice across the globe, yet much can be done to optimize its beneficial impact. Taking advantage of pivotal opportunities to enhance this approach will require engagement with stakeholders, including government, funding agencies, and the general public, on: (a) the need for greater precision in our understanding of how current neonatal vaccines protect, the potential impact of the exact timing of administration in the neonatal period (i.e., first 28 days of life) and of vaccine–vaccine interactions, (b) assessing how maternal and neonatal immunization can be best integrated, and (c) leveraging modern tools including systems biology and human *in vitro* modeling to study the impact of immune ontogeny on vaccine responses thereby informing development of novel vaccines for use in early life against pathogens for which currently vaccines are inadequate (e.g., pertussis, TB, and influenza) or do not yet exist (e.g., RSV, HIV).

## Author Contributions

EW and OL outlined the initial manuscript drafts; EW, OL, PM, and DB all contributed to the final manuscript.

## Conflict of Interest Statement

The authors declare that the research was conducted in the absence of any commercial or financial relationships that could be construed as a potential conflict of interest.
